# Back to the future? Reflections on three phases of education policy reform in Wales and their implications for teachers

**DOI:** 10.1007/s10833-021-09422-6

**Published:** 2021-03-23

**Authors:** Gareth Evans

**Affiliations:** Yr Athrofa: Centre for Education, University of Wales Trinity Saint David, Swansea, SA1 8EW Wales, UK

**Keywords:** Accountability, Curriculum, Education policy, Professional learning, System reform, Teacher agency

## Abstract

Wales’ education system is part-way through an extensive journey of reform. This contextual paper explores the evolution of that journey, from the establishment of the Welsh Parliament in 1999 to late 2020, as Wales readies itself for the launch of a radical, new national curriculum. Drawing from a range of international literature and experience, it provides an overview of key policy developments and insight into the rationale for decisions taken by the Welsh Government to effect change. To do this, it separates reform into three core phases, each with its own characteristics borne out of landmark events that helped shape contemporary political and public discourse. In particular, the paper examines the impact of Wales’ shifting approach to policy development on the teaching workforce and considers implications for those at the site of practice. Ahead of forthcoming parliamentary elections, the paper resolves that a new, long-term approach to policy reform and teacher development is needed if Wales is to realise its ambitious vision for education.

## Introduction

Wales is a relatively small country that is a constituent part of the wider United Kingdom (UK), a socially and politically complex island nation that gives each of its member countries the authority to administer and develop their own public services. With around 3.1 million inhabitants, Wales has sought to preserve its distinct cultural identity and now enjoys a form of self-government via Senedd Cymru, the Welsh Parliament. Created in 1998 (and known as the National Assembly for Wales until early 2020), it sat for the first time a year later and is the country’s democratically elected body, with power to make legislation, vary taxes and scrutinise the ruling Welsh Government. An incremental devolution of powers from the UK Parliament in Westminster to Senedd Cymru in Cardiff Bay has given Wales almost total control over key policy areas including health, economic development, transport, the environment and education. The Welsh Government is thus responsible for the oversight and delivery of education across *all* stages, despite a level of decentralisation to local authorities and their four supporting regional education consortia (a relatively new entity allocated school improvement functions in 2012). This strengthening of responsibility has allowed policymakers in Wales to pursue a discrete strategic policy on education, resulting in greater divergence between itself and the other ‘home nations’ of England, Scotland and Northern Ireland. Indeed, given the contrasting political agendas of the UK and Welsh governments (the Welsh Labour Party having been in power in Wales since 1999, unlike in England where the Conservatives have governed either on their own or in coalition since 2010), this position appears very likely to continue. The subsequent fracturing of education systems across the UK has given rise to the narrative, espoused by successive Welsh education ministers, that there is an alternative ‘Welsh way’ to policy design and implementation. This paper charts the evolution of Wales’ reform journey and separates education policy development into three core phases (Connolly et al. 2018; Egan 2017), each with its own characteristics borne out of landmark events that helped shape contemporary political and public discourse. In so doing, it challenges the notion that education since devolution has been linear in its construction and while there are noteworthy similarities between phases, so too are there marked differences. The paper identifies a number of recurring features, revitalised under different educational leadership, and provides new insight into the factors contributing to what could be considered a *disjointed* series of reforms. It addresses each phase in turn and resolves that all three had significant and far-reaching implications for the education workforce in Wales, which has withstood and adapted to a raft of subsequent changes. The paper concludes with an evaluation of the Welsh approach to education reform and offers a possible way forward for policymakers, based on lessons learned from two decades of devolution.

## Welsh context

In January 2020, there were approximately 469,000 pupils in Wales, in nine nurseries, 1,225 primary schools, 22 middle schools (which include both primary and secondary education), 183 secondary schools and 41 special schools (Welsh Government 2020a). There are no ‘free schools’ or academies in Wales as there are in England and schools are instead funded by 22 local authorities, which receive the majority of their funding through the Welsh Government’s annual local government budget. The country is officially bilingual and around 450 schools identify as teaching predominantly through the medium of Welsh, which is compulsory for all pupils regardless of first language up to the age of 16. Indeed, the growth of the Welsh language is considered a relative success story and an ambition to boast a million Welsh speakers by 2050 is a national priority (Welsh Government 2017a). As of March 2019 there were around 35,500 school teachers registered in Wales, which represents a reduction of 4.8% since 2015. The majority of teachers are female (75.5%) and there has been a gradual decrease in the proportion of male teachers from 28.1% in 2002 to 24.5% in 2019, while three-quarters (75%) of teachers are aged under 50. Around a third of teachers are Welsh-speaking (33.3%) and in the secondary sector there are more teachers trained specifically in English and mathematics than in any other subjects (EWC 2019). Retention rates are an ongoing concern and while the number of teachers leaving the profession has fallen overall since 2007, there has been a notable increase in the number of teachers leaving since 2012 and 814 teachers left the profession in 2016 alone (Beaufort Research 2019). Outside of retirement, teachers in Wales with less than five years’ experience are the most likely to leave. All of this has contributed to the perception that the country’s education system faces a ‘crisis’ in recruitment to some positions (Connolly et al. 2018).Wales employs a comprehensive school system that promotes equity and inclusion, yet faces serious issues regarding inequality; almost a third of children live in poverty and its proportion of employees who are the lowest-paid is the highest in the UK (Save the Children 2019). The attainment gap between children of different social backgrounds is a perennial focus for ministers, with the performance of disadvantaged pupils at Key Stage 2 (age 11) around 14 percentage points lower than those who are less disadvantaged (Joseph Rowntree Foundation 2019). The picture is similar at Key Stage 4 (age 16), where pupils eligible for free school meals (a proxy for deprivation) achieve approximately 31 percentage points fewer than their more affluent peers (ibid). Overall, the poverty rate has been higher in Wales than for England, Scotland and Northern Ireland in each of the last 20 years (Barnard 2018).

## Phase one: Devolution and a licence to innovate (1999–2010)

The advent of devolution and the shifting of responsibility for the vast majority of matters pertaining to education to a new national legislature in Cardiff Bay brought with it a newfound licence to innovate, and it is in this context that I propose *Phase One* of Wales’ policy reform journey begins. Liberated from Westminster rule, Welsh ministers appeared to revel in the opportunity to plough their own furrow and do things differently to England (which is wholly governed by the UK Government, unlike Wales) in the decade from 1999–2010. This is, perhaps, best demonstrated in former First Minister Rhodri Morgan’s famous ‘clear red water’ speech, setting out how the Welsh arm of the Labour Party would seek to differentiate from Prime Minister Tony Blair’s Labour Party in London. Indeed, Morgan (2002) was confident that a great deal had been achieved in turning the former Welsh Office, which preceded the then Welsh Assembly Government (WAG—now Welsh Government), ‘from an engine of administration into one which analyses and develops policy choices’. A number of the Welsh Government’s most prominent policy choices can be traced back to *The Learning Country* (WAG 2001), which outlined the administration’s 10-year vision for education and lifelong learning in Wales. In it, the document’s principal author, long-serving Education Minister Jane Davidson, noted that while Wales shared key strategic goals with colleagues in England, ‘we often need to take a different route to achieve them’ and ‘we shall take our own policy direction where necessary, to get the best for Wales’ (WAG 2001, p. 2). A more impartial view of the WAG’s strategy is offered by education historians Jones and Roderick (2003, p. 124), who described *The Learning Country* as a ‘landmark document for those who hoped that the Welsh Assembly would not just nibble at the edges of educational policy-making but would also conjure up a wider vision of an education system to serve the Welsh nation’.

*The Learning Country* brought with it a number of policy innovations that separated very clearly Wales’ education system from that in England. This divergence, owing in part to what Power (2016, p. 287) considers the countries’ ‘contrasting orientations to traditionalism and progressivism’, resulted in a different approach to curriculum and assessment based on the enabling of practitioners to support children’s ‘rounded progress’, as opposed to focussing more acutely on academic, standards-based frameworks (WAG 2001, p. 20). The most eye-catching example of Welsh progressivism in education was the introduction of the ‘Foundation Phase’, a flagship early years policy that adopted a ‘developmental’ approach to children’s learning (Maynard et al. 2013, p. ix). Built on the principles of ‘learning through play’ (Welsh Government 2018), the Foundation Phase was based on the Scandinavian model of child-centred education and would have significant implications for the professional workforce in Wales. Indeed, shortly after the policy’s introduction in pilot form in 2004, Siraj-Blatchford et al. (2005) warned that the WAG would need to make clear its commitment to raising standards and the quality of educational provision by improving the qualifications and training of staff. Their independent review suggested that those working within the Foundation Phase ‘need to be better qualified and trained to work with young children effectively and appropriately’ and ‘a national training policy needs to be implemented to ensure that staff have the necessary expertise to undertake this work’ (Siraj-Blatchford et al. 2005, p. 80). A raft of evidence published some years later as a part of an extensive evaluation of the Foundation Phase (WISERD 2013), which was made statutory from 2010, suggests that insufficient direction and support at a national level hampered its development. Other innovations, leading to a further break-up of the interconnected education systems of Wales and England, included the *Curriculum Cymreig*, which encouraged the teaching of a ‘Welsh dimension’ to help pupils understand the distinctive quality of living and learning in Wales, and to further their sense of belonging to their local community and country (ACCAC 2003). Fuelled by a newfound political patriotism, the *Curriculum Cymreig* was symptomatic of the WAG’s attempt to claw back its national identity by ensuring a strong focus on developing pupils’ knowledge, skills and understanding within a specifically Welsh context (ibid 2003). Moreover, it allowed Wales to differentiate more easily its curriculum from the curricula found in other home nations (Smith 2016). But the first decade of devolution can be remembered as much for what was dropped as that which was introduced.

### Decentralising power and responsibility

A very noticeable rowing back from established accountability mechanisms saw secondary school league tables scrapped in Wales in 2001 and Standardised Assessment Tests (SATs), for pupils aged seven, 11 and 14, phased out between 2002 and 2005. For some, such a move represented a ‘radical departure from the English vision’ (Jones and Roderick 2003, p. 124) but for others, like Reynolds, it was reflective of the WAG’s ‘attempt to get education by consensus, to generate education change by being nice to people rather than being nasty’ (Archer 2008). For teachers in Wales, the first decade of devolution would bring forth contrasting challenges, dependent largely on the sector in which they operated. For those working in primary or early years settings, the implementation of the Foundation Phase and with it, the introduction of an entirely new curriculum for three to seven-year-olds, would necessitate the adoption of particular pedagogical practices (Taylor et al. 2016). In the secondary sector, the Welsh Baccalaureate, introduced between 2003 and 2007 to provide young people with a broader skills base ready for further study and employment, added another layer to what was already deemed a ‘broad and balanced curriculum’ (WAG 2001, p. 61). An emphasis on the ‘informed professional judgement of teachers’ as one of *The Learning Country*’s key guiding principles, coupled with a commitment to unleashing the capacity and expertise of practitioners, was indicative of the WAG’s desire to shift responsibility for education more clearly to the site of schools (WAG 2001, p. 11). This decentralising of power to education’s frontlines was in stark contrast to future approaches and, together with the raft of new initiatives introduced by Davidson, contributed to the shaping of a uniquely ‘Welsh’ education system. Jane Hutt, who succeeded Davidson as education minister in 2007, continued her predecessor’s commitment to the Foundation Phase by revising the school curriculum to include a non-statutory ‘Skills Framework’ and ‘14–19 Learning Pathways’, a strategy designed to offer learners a fuller range of academic and vocational options. Somewhat appropriately, these developments were initiated within the remit of *One Wales* (WAG 2007), the ‘progressive agenda’ of coalition partners Welsh Labour and Plaid Cymru, who held power in the National Assembly for Wales (now Senedd Cymru) from 2007–2011. Nevertheless, the appointment of Leighton Andrews as education minister, together with the publication of an influential benchmark of Welsh performance, would elicit a noticeable change in approach.

## Phase two: PISA and the age of accountability (2010–2015)

The Programme for International Student Assessment (PISA) tests taken by teenagers across the world in 2009 and published a year later in 2010, had a considerable impact on Wales’ school system and can be considered a major turning point for Welsh education post-devolution. A measure of the knowledge and core skills of 15-year-olds as they near the end of their compulsory education, PISA showed student performance in Wales to be significantly below the rest of the UK and the Organisation for Economic Co-operation and Development (OECD) average in reading, mathematics and science (NFER 2010). The attainment of Welsh 15-year-olds in all three domains was lower than that recorded previously in 2006 (when Wales participated in its own right in PISA for the first time) and Leighton Andrews, who replaced Hutt as education minister in late 2009, described the scores as a ‘wake-up call to a complacent system’ (Dauncey 2016). Calling for collective responsibility, he said:

These results have made it clear that schools in Wales are simply not delivering well enough for students at all levels of ability. There can be no alibis and no excuses. Countries with less money spent on education have done better than Wales. Schools, local authorities and ourselves as government need to look honestly at these results and accept responsibility for them. (Andrews 2010)

In a highly-charged address to the nation’s education workforce in early 2011, Andrews said he regarded PISA a ‘highly respected and robust measure of the relative performance of educational systems’ and Wales needed to ‘face up to the harsh truth’ that it was not delivering the outcomes that young people deserved (Andrews 2011). Although powerful in tone, Andrews’ response was not completely unexpected given the perceived severity of Wales’ results and while some considered it an ‘overreaction’ (Reid 2013), his words were comparable to those used in other jurisdictions following the publication of disappointing PISA scores (see e.g. Baroutsis and Lingard 2018; Ertl 2006). Indeed, Wales was not alone in using PISA to justify policy change and the country’s political establishment simply mirrored what many other countries had done in its acceptance of PISA as a proxy for school system performance (Breakspear 2014; Sahlberg 2011; Takayama 2008). The national response to PISA can be likened to that experienced by Germany in 2001, when the publication of results had a ‘Tsunami-like impact’ on educational policy-making and discourse (Gruber 2006). In Wales, a nationwide phenomenon of ‘PISA shock’ (Waldow 2009) made the public more aware of large-scale assessments taken internationally and its coverage in Welsh newspapers and the wider media brought the issue of school standards to the fore. The portrayal of Wales’ education system as having ‘underperformed’ and, indeed, ‘gone backwards’ since the nation’s first foray into PISA contributed to the narrative that ‘something had to be done’ to arrest the perceived decline in pupil performance. In so doing, Wales joined the growing list of countries that sought to respond very seriously in political and policy terms to PISA shocks (Baroutsis and Lingard 2018). It is with Andrews’ appointment and subsequent fallout from PISA in mind that I propose a swift transition to *Phase Two*.

In accepting PISA as a valid measure of pupils’ basic skills, as well as the education system’s capacity for improvement, Andrews was able to authenticate Wales’ educational failure relative to other nations and leverage wide-ranging reform on the back of it. The fact he regarded PISA ‘a highly respected and robust measure’ (Andrews 2011), despite growing evidence to the contrary (Bautier and Rayon 2007; Goldstein 2004), cemented its positioning within wider public discourse and there was no discernible reference to the known variation in administration and analysis of PISA data (Jerrim et al. 2018) to counteract these claims. Indeed, the propensity of governments to use PISA as a key source of education policy development, and the reason for many large-scale education reforms (Sahlberg 2011), is entirely relevant here as popular debate in Wales almost wholly ignored PISA’s limitations, treating it instead as if it were entirely definitive (Rees and Taylor 2015).

### Sharper focus on outcomes

Wales’ failure to improve its international standing relative to other PISA jurisdictions, most notably those in close proximity (e.g. England), resulted in what one union leader described as a ‘white-knuckle ride for education’ (Evans 2015, p. 37). This involved the unveiling of a slew of new initiatives with a renewed emphasis on standards and higher expectations across every aspect of the education system. The Welsh Government’s ‘20-point plan’ (Andrews 2011) was centred around literacy and numeracy, as well as greater accountability and stronger leadership in schools. It included the creation of a ‘School Standards Unit’ within the Welsh Department for Education to ensure a sharper focus on outcomes; greater emphasis on the use of data to track pupils’ progress; and the launch of a new master’s-level qualification to better support new entrants to the teaching profession. A pledge to integrate PISA assessments into school assessment at age 15 was designed primarily to ensure buy-in from teachers required to work with pupils during the next PISA tranche. There was, to some extent, a clamour for data, owing at least in part to what Andrews believed was a lack of available information about how schools were performing. This derived from a perceived cosiness between stakeholders, and a reluctance during the first phase of devolution to ‘rock the boat’ (Andrews 2011). To combat what Andrews considered a *cultural* weakness, the minister took steps to embed ‘best practice’, warning those who refused to ‘adopt or justify’ (ibid). The introduction of ‘robust self-evaluation and rigorous benchmarking’ would, he said, mean ‘that all schools can learn from the best in class’ (Andrews 2011). Together, these interventions fuelled the emergence of what can be considered a performativity culture, ‘where knowledge is perceived as measurable and often explicitly defined’ (Hennessy and McNamara 2013, p. 9).

Two of the more notable policy developments post-PISA related to the revival of national testing and the re-introduction of a public-facing school ranking mechanism. Launched in 2012, a new national ‘banding’ system (known now as the *National School Categorisation System*) would rank every primary and secondary school in Wales annually using a ‘traffic-light system’ to identify ‘the schools that need the most help, support and guidance to improve’ (Welsh Government 2019a). National Reading and Numeracy Tests for all pupils aged seven to 14 were introduced a year later to provide a national picture of how pupils were performing and make it easier for teachers to identify strengths and weaknesses. Since their inception, both categorisation and national testing (which remain important features of the Welsh Government’s standards agenda) have courted controversy, not least because of their similarity to SATs and secondary school league tables, scrapped in Wales during the early 2000s (Evans 2015). Moreover, it could be argued that categorisation and national testing were in fact variations on a theme and Andrews’ decision to revive them merely a stealthy undoing of the work of his predecessor. While policy reversal of this ilk is not uncommon (Hood 1994), what is notable in this context is that Andrews, a Welsh Labour politician, was of the same political leaning as each of his preceding four Welsh education ministers.

In some respects, the reintroduction of two new layers of accountability into Wales’ education system might have been intended to re-position public sector schools as competitors in the marketplace, thereby encouraging them to behave more like those in the private sector (Whitty 2006). However, while justified in terms of providing more information to the ‘consumer’, there was a general understanding that the revival of such indicators would better enable government to scrutinise and direct providers of education against pre-determined outcomes. Regardless of the minister’s intentions, the changing nature of categorisation and national testing was symptomatic of the turbulence and volatility that existed within the system at the time. Indeed, Andrews’ ambition for Wales to rank within the top 20 of all PISA participants by 2015 was as much a hurried and impulsive response to Wales’ 2009 results (he would later regret setting such a lofty forecast), as it was a reflection of the strong political mandate he had been given to effect change (Andrews 2014). The notion of ‘school improvement’ would become the cornerstone of education policy in Wales during Andrews’ three-and-a-half-year term as education minister (Rees and Taylor 2015), albeit there was some concern that the breadth of the minister’s interventions was too vast and not concentrated enough (Evans 2015). The strain of the Welsh Government’s weighty reform agenda on the education system itself was highlighted in a much-referenced country review, conducted by the OECD in 2014. Promising lessons from ‘PISA high-performers and successful reformers’, the OECD warned that teachers in Wales had been ‘overwhelmed’ by the pace of change and the nation lacked a long-term vision (OECD 2014, p. 66). Furthermore, it suggested that the challenge for Wales did not lie in a lack of willingness and commitment of the profession to implement desired policy, but rather with the time and resources they had been given to do so (ibid 2014). Cognisant of the ‘sense of urgency’ borne out of Wales’ poor PISA results in 2009, and further disappointment in 2012 (when Wales was again ranked below the rest of the UK in all three PISA disciplines), the OECD argued that stakeholders had no clear understanding of the country’s long-term goals beyond its aspiration to be among the 20 best-performing education systems by 2015. More specifically, it said:

The evidence suggests Welsh schools are currently facing some challenges in implementing the numerous policies and reforms… particularly because there are so many. The head teachers and other stakeholders that the OECD review team met felt that the sheer number and often short time spans for schools to implement these reforms bring with them a risk of only partial implementation of reforms, or ‘reform fatigue’. (OECD 2014, p. 34)

The OECD recommended a number of concrete policy options designed to strengthen Wales’ education reform and help ensure schools appropriately met the learning needs of all their pupils. Mindful of the need to develop an adequate school improvement infrastructure and a clear implementation strategy that all stakeholders shared, the OECD argued for the introduction of a long-term plan, translated into measurable objectives. It resolved that ‘a shared longer-term vision of the Welsh learner and the education system at large that could evoke the intrinsic motivation of those involved and further focus the reform journey is lacking’ (OECD 2014, p. 116).

### Comparisons with England

While the second phase of education policy development in Wales was noticeably more draconian than the first, and displaying of a number of neoliberal characteristics commonly associated with data-driven accountability systems (Liasidou and Symeou 2016; Waitoller and Kozleski 2015), the extremity of its dominant neighbour’s approach to quality assurance and more hostile portrayals of the teaching profession (Power 2016) ensured the continuation of a wedge between its own educational agenda and that being pursued in England. The Westminster Government’s commitment to high-stakes school accountability (West 2010) and a more deliberate ‘governing by numbers’ (Grek 2009) made Wales’ tightening of performance measures appear lighter touch, by comparison. Indeed, the delegitimising of Welsh education policy by newly-appointed Conservative ministers in England and a squeezing out of alternative conceptions of what constitutes educational ‘success’ (Power 2016) all but guaranteed a separation of their respective education systems. Under Andrews, the approach to policy reform could be considered something of a hybrid in that it retained the progressive features of previous left-wing administrations, yet at the same time reintroduced the categorising of schools, national testing and use of data to drive performance. The middle phase (*Phase Two*) of Welsh education’s evolution under Welsh Labour was underpinned by a commitment to equity of opportunity, comprehensive schooling and the burgeoning Foundation Phase, but it was striking in its flagrant disregard for the rowing back from accountability championed just a short while earlier. Indeed, while the ‘back to basics’ approach (Power 2016) adopted by Education Secretary Michael Gove in England was more openly regressive, there were common characteristics between that and Andrews’ modus operandi, particularly with regards to their expectations of schools and the way in which they were held accountable. It could be argued that the growing pressure on education systems to align what they do ‘with the alleged imperatives of the global economy’ (Rizvi and Lingard 2011, p. 18), coupled with the desire to improve performance based on an ‘accountability culture of outcomes’ (Tatto 2012, p. 2), ensures at least a level of congruence between countries pursuing the same goals: namely, to compete internationally on the world stage. As Ball (2013) suggests, a preoccupation with quality indicators, target-setting and measurement means national education reform agendas often share performative similarities. It is perhaps not surprising, therefore, that such similarities are evident in the approaches taken by Wales and England during this time.

## Phase three: Curriculum for Wales and a culture of collaboration (2015–)

The OECD considered the publication of its *Improving schools in Wales* report (2014) an opportune time for a renewed education strategy, involving teachers and other key actors, that would sequence the development and implementation of various initiatives and take account of the system’s capacity to support this activity. The Welsh Government took heed of the OECD’s advice and in 2014 launched *Qualified for Life* (Welsh Government 2014), its five-year education plan for three to 19-year-olds in Wales. Built around four strategic objectives, the document sought to develop ‘an excellent professional workforce’ with a strong focus on pedagogy; an engaging and attractive curriculum; credible and internationally-respected qualifications; and a self-improving system, involving leaders of education at all levels working together to raise standards (ibid, p. 5).The publication of *Qualified for Life* was overseen by Huw Lewis, who followed Andrews as education minister in 2013 and remained true to the Welsh Government’s overarching priorities of strengthening literacy and numeracy and breaking the link between disadvantage and educational attainment. Lewis is, perhaps, best remembered for inviting Graham Donaldson to review Wales’ curriculum and assessment arrangements, which later manifested in the publication of *Successful Futures* (Donaldson 2015). An international advisor to the OECD and former chief inspector of education in Scotland, Donaldson was instrumental to the development of the Scottish *Curriculum for Excellence* (Scottish Executive 2004), which would bear a number of similarities to the curriculum outline published in Wales. *Successful Futures* presented 68 recommendations, including that a new *Curriculum for Wales* be based on Four Purposes and structured around six Areas of Learning and Experience. Encompassing everything from the Foundation Phase to Key Stage 4 (i.e. the entire age range from three to 16), the curriculum would champion literacy, numeracy and digital competence as cross-curricular responsibilities that would be the domain of all teachers, regardless of subject or age specialism. *Successful Futures* aimed to develop children and young people as: ambitious, capable learners, ready to learn throughout their lives; enterprising, creative contributors, ready to play a full part in life and work; ethical, informed citizens of Wales and the world; and healthy, confident individuals, ready to lead fulfilling lives as valued members of society (Donaldson 2015). Chiefly, the report argued for a continuum of learning rather than the separation of schooling into key stages as was traditionally the case, and sought to reassert the fundamental purposes of education for the betterment of all children and young people in Wales. The curriculum would have significant implications for teaching and necessitate a sound understanding of cross-cutting themes, new curriculum areas (e.g. Health and Wellbeing), the Welsh language and effective assessment practices. By accepting in full Donaldson’s recommendations, Lewis had effectively marked the beginning of a new era in policy development—*Phase Three*—that would define Wales’ education system from mid-2015 to the present day. Later described as the ‘cornerstone’ of the country’s efforts to move from a performance-driven education system to one more clearly focussed on the learner (OECD 2020), the new curriculum would herald a softening in neoliberal ideology and a more collegiate approach to policy development. Indeed, Lewis (2015) himself noted that ‘the sustained and active participation of educational practitioners and the wider community’ would be essential to the curriculum’s successful implementation.

### The ‘Pioneer’ model

The need to involve practitioners in the curriculum development process, as alluded by Lewis, was apparent in the collaborative ‘Pioneer’ model employed by the Welsh Government to drive change. A network of ‘Pioneer Schools’ was established in late 2015 to spearhead the curriculum project and included schools from both urban and rural communities and a mixture of English-medium and Welsh-medium, primary and secondary, and special and faith schools. Education unions commended the approach as recognition of the expertise education professionals could bring to policy development, and their ‘critical’ role in ensuring the new curriculum was properly implemented (Evans 2015). The Pioneer model was innovative in the Welsh context and had at its core a commitment to both empowering and supporting teachers to develop their own practice and that of others (Arad Research 2018). It was welcomed as a distinctly new and positive way of working, and its methods of co-construction were in stark contrast to the more ‘top-down’ (Bishop and Mulford 1999) and centrally-driven approach to policymaking of which teachers had become accustomed (OECD 2017). This was a deliberate undertaking, recognising that collaborative practices of this nature shift the drive for improvement away from government to the front lines of schools, helping to make them more self-sustaining (Barber et al. 2010). Built on the assumption that participating schools would *pioneer* their way through a challenging curriculum landscape and plot a course for their colleagues in other schools to follow, the Pioneer concept was indicative of both the growing international emphasis on schools working together to raise standards and a professional belief in the efficacy of locally-generated solutions to shared challenges (Coleman 2012; Hadfield and Ainscow 2018; Kidson and Norris 2014). Indeed, the curriculum itself marked a radical departure from the ‘teacher proof policy’ of previous national curricula and afforded teachers considerable autonomy to develop programmes of learning to meet local needs (Sinnema et al. 2020). Early evaluations of the Pioneer model suggest that school representatives—senior managers and other practitioners engaged in the process—were enthusiastic about their experiences of being involved in the curriculum reform journey, noting the ‘clear sense of ownership and responsibility’ associated with being a Pioneer School (Arad Research 2018, p. 6). Researchers highlighted the ‘multi-faceted and complex’ nature of the approach, that required teachers taking ownership of the curriculum’s development within a national framework of support (ibid, p. 6). For Donaldson, the perception that teachers had been constrained by existing practices provided further justification for change. He noted:

The high degree of prescription and detail in the national curriculum, allied to increasingly powerful accountability mechanisms, has tended to create a culture within which the creative role of the school has become diminished and the professional contribution of the workforce underdeveloped. (Donaldson 2015, p. 10)

In this respect, *Successful Futures* represents one of several curricula being developed across the world to *guide* educational practice, rather than as a prescriptive recipe to be followed to the letter (Drew and Priestley 2016). Indeed, the building of teacher ‘agency’ and with it professional autonomy, was central to Donaldson’s (2015, p. 71) vision for an education workforce with a sound understanding of the ‘why’ and ‘how’ of teaching as well as the ‘what’. However, Donaldson would acknowledge that the implications for the formation and subsequent growth of teachers as reflective practitioners were considerable and if the proposals outlined in *Successful Futures* were to be realised, there would need to be an extensive, well co-ordinated and sustained professional learning programme involving all leaders, teachers and other practitioners (ibid).

### A renewed vision for education

*Qualified for Life* was revised in 2017 following the appointment of Kirsty Williams as education minister. A Welsh Liberal Democrat in a minority Welsh Labour government, Williams adopted many of its prominent features and remained true to *Successful Futures*, recognising that Wales’ education system was only part-way through the reform journey mapped out under Huw Lewis. *Education in Wales: Our National Mission* (Welsh Government 2017b) was strikingly weightier than its preceding document (running to almost double the amount of pages) and made clear that the building of a new curriculum would underpin all of Wales’ change agenda. Indeed, the title of the document itself was reflective of a deliberate attempt by the Welsh Government to make clear its new four-year educational vision and respond to criticism levelled earlier by the OECD (2014). Despite its prominence in all strategic documentation, the Welsh Government has since conceded that curriculum reform will not by itself bring about the level of change required to deliver for Wales’ children and young people. As such, policymakers have embarked on a process of ‘whole-system’ reform, described by Fullan (2010) as involving every part of the education workforce—schools, communities, local authorities and government—contributing individually and in concert to forward movement and success. Thinking of whole-system reform as ‘a means of focusing on a small number of core policies and strategies, doing them well as a set, and staying the course by not being distracted’ (Fullan and Levin 2009) is useful in this regard, as it helps separate the Welsh Government’s new approach to policy development from that it had employed previously. There is certainly a perception, widely held in Wales, that the reforms introduced immediately following the publication of PISA results in 2010 were delivered *to*, rather than *with*, the education workforce (Connolly et al. 2018) and thus *Education in Wales: Our National Mission* recognised the need for a more collaborative, sustainable and integrated approach to policy development. In that respect, the document marked a sea change in how education policy would be shaped in Wales.

Williams (2019) has described Wales’ current reform agenda as the biggest anywhere in the UK for over half a century and quotes Dalton McGuinty, the former Liberal Prime Minister of Ontario, when asserting that ‘government’s commitment to learning is the single-most important thing we can do for our future’. A self-confessed social reformer, Williams is a firm believer in Wales’ non-selective comprehensive school system and that nobody’s background should determine their future. Her strong commitment to matters of social justice can be evidenced in the Welsh Government’s ongoing and substantial investment in the Pupil Development Grant, a £94 million-a-year fund that provides extra money to schools based on their number of pupils eligible for free school meals (National Assembly for Wales 2018). Since her appointment, Williams has led the introduction of a new *National Academy for Educational Leadership*, revised professional standards for teachers and leaders, reformed initial teacher education provision, and unveiled a new *National Approach to Professional Learning*. Moreover, the perceived negative effects of a ‘high-stakes’ accountability culture (Jones and Tymms 2014), involving the reporting of outcomes in national tests and using the performance of pupils in public exams to categorise schools, has contributed to lengthy discussion regarding the way in which schools are held accountable for the service they provide. Among topics for debate has been the application of this data by external agencies, known to use the same information at different times and for different purposes (Connolly et al. 2018). There are fears that teachers’ moral obligation to do the best by their pupils has, at times, been challenged by the need to ‘play the game’ (Gleeson and Gunter 2001). Under Williams, the Welsh Government has ended the routine publication of teacher assessment data and national test results, and sought to reduce the weight of outcome indicators on school categorisation. In addition, and as part of the minister’s attempt to develop a new school accountability system fit for the needs of the forthcoming curriculum, an independent review of the nation’s education inspectorate, Estyn, was commissioned and found that benchmarking tools such as those currently used ‘can inculcate a culture of fear, inhibiting creativity and genuine professional analysis and discussion’ (Donaldson 2018, p. 23). New inspection arrangements based around self-evaluation and professional dialogue have since been proposed, and a partial suspension of school inspections to enable Estyn and its stakeholders to engage in curriculum preparations agreed. Table 1 outlines the landmark events and publications in Wales’ education policy reform journey since devolution, ending with the release in early 2020 of *Curriculum for Wales* steering documents.

**Table 1:** Landmark events/publications in Wales’ education policy reform journey since devolution

Year	Event/publication
1999	National Assembly for Wales sits for first time
2001	*The Learning Country* published; Secondary school league tables scrapped
2002–2005	SATs, for pupils aged seven, 11 and 14, phased out
2003–2007	*Welsh Baccalaureate* rolled out
2004–2005	*Foundation Phase* piloted
2010	2009 PISA results published
2011	*‘20-point plan’* unveiled
2012	New school ‘banding’ system introduced
2013	*National Reading and Numeracy Tests* introduced
2014	*Qualified for Life* published
2015	*Successful Futures* and *Teaching Tomorrow's Teachers* published
2017	*Education in Wales: Our National Mission* published
2020	*Curriculum for Wales* framework unveiled

A significant reimagining of initial teacher education in Wales was prompted by *Teaching Tomorrow’s Teachers* (Furlong 2015) and considered integral to realising the Welsh Government’s ambitions, as outlined in its *National Mission*. A comprehensive professional learning programme is now being crafted alongside teacher education reforms, which launched in 2019, to ensure all school leaders and teachers in Wales ‘have the skills, capacity and commitment to continually learn and improve their practice so that every child achieves their potential and is prepared for life in an increasingly complex world’ (Welsh Government 2017b). It has doubtless been recognised that only ‘a well-respected teaching profession’, which is supported in its professional learning, will ensure that quality student learning outcomes are achieved (Sachs 2007, p. 18). This and issues relating more specifically to the teaching profession will be considered in greater detail in the following section.

## What next for Wales?

Linked by a fundamentally different conception of what it means to be a practitioner in Wales, many of the policy interventions explored in *Phase Three* and being embedded currently are almost entirely reliant on the capacity and capability of teachers. Indeed, the contemporary approach to education in Wales places teachers at the heart of the reform process, in that they will contribute meaningfully to each stage of policy development—from initial conception right the way through to implementation. This is at odds with approaches taken elsewhere in the UK (e.g. England) and is in direct contrast to the more regressive approach taken in Wales a decade ago. From a system characterised by punitive, high-stakes accountability and challenge (*Phase Two*) has evolved a very different system built on subsidiarity and trust. In practice, this has meant a quiet erosion of the Welsh Government’s intensive focus on quantitative performance measures to assess progress, with more attention being paid to qualitative sources. By allowing teachers greater autonomy over their day-to-day practice, Welsh ministers have effectively instigated a process of decentralisation from the civil service down to schools. There is broad consensus that this transition will require a new ‘type’ of teacher and new ways of working, albeit that the desired evolution of the teaching profession in Wales will not come easily and necessitates a shift in culture. This shift, however, rests initially upon an awareness on the part of the teacher to ‘be aware of, and knowledgeable about, the consequences of different choices of content and methods’ (Englund 1996, p. 83), thus recognising their own limitations and potential growth areas. It is likely that the development of teachers as competent, thoughtful, reflective and innovative practitioners who are ‘always learning, improving and researching so that they develop and adapt’ (Williams 2017) will be reliant on their developing a new ‘habit of mind’ (Hill 1997). Defined by Costa and Kallick (2000) as ‘broad, enduring and essential lifespan learnings’, habits of mind that are conducive to and accepting of change will be of huge significance here, as Bandura’s (1997, p. 3) seminal work on teacher self-efficacy, and the ‘beliefs in one’s capabilities to organize and execute the courses of action required to produce given attainments’ testifies. Similarly, if one conforms to the definition of agency as a teacher’s ‘capacity to make choices, take principled action, and enact change’ (Anderson 2010, p. 541), then so too must we acknowledge its temporal dimension as something occurring over time (Biesta et al. 2017, p. 40). Emirbayer and Mische’s (1998) conceptualisation of agency as a temporally embedded process of social engagement, informed by the past but also oriented toward the future as well as the present, is relevant here as it recognises the myriad of experiences impacting on teacher development. Jenkins (2020) argues that teacher agency can be manifested in a combination of three ways: as *proactive* agency, whereby teachers plan for and initiate curriculum change as a personal choice; as *reactive* agency, involving teachers making changes in response to an environmental influence; and as *passive* agency, where teachers passively resist a required curriculum change yet may have appeared to their leadership to have implemented it. In the case of Wales, it is reasonable to surmise that there are teachers displaying characteristics of all three manifestations. This in some part is a consequence of the ‘Pioneer’ model, which by design creates a disparity in understanding of reforms based on the participation of individual teachers from selected schools. This in and of itself presents a formidable challenge, and ensures a ‘one-size-fits-all’ approach to professional support will not be sufficient in meeting the needs of all key stakeholders. Ultimately, one is mindful that ‘like anywhere else, Wales’ new curriculum and assessment arrangements can only be successfully implemented if the teachers, teaching staff and other actors of learning have the adequate capacity to turn the policy into reality’ (OECD 2020, p. 43). It is with that in mind that I propose a *revitalising* of the education workforce is required.

### Revitalising the education workforce

There is little doubt that the culture shift required of Wales’ education workforce to make good the nation’s ongoing reform agenda is significant, and from passive consumers teachers will need to become proactive producers of curriculum content. Yet Wales finds itself in a precarious position, balancing on the one hand a newfound ‘sense of ownership and empowerment where workers aim to grow within their profession and seek increased responsibility’ (Dondero 1997, p. 218) with, on the other hand, ‘this problem of ensuring that the teachers have the leeway, the time, the foresight and competence to introduce new ideas’ (Jónasson 2016, p. 9). It is well-documented that education systems are themselves responsible for nurturing or stifling teacher agency (Biesta et al. 2015; Sachs 2015), and that ‘sustained and effective development of new knowledge and competencies within the teaching profession needs an effective system, anchored also within the schools and their practices’ (Jónasson 2016). In this context, the Welsh Government’s *National Approach to Professional Learning*, which promotes ‘a professional learning vision fit for the evolving education system in Wales’ (Welsh Government 2019b), appears the most likely vehicle through which professional support for the education workforce can be achieved, though it is unlikely that this framework alone will be enough to transform practice in every classroom. In order to revitalise the education workforce in preparation for the new curriculum, it could be argued that Wales requires a new culture of ‘informed professionalism’ (Barber 2001), or what Noordegraaf (2007) considers a process of ‘reprofessionalisation’ involving the redefining of teachers’ working roles to meet shifting expectations. Both of these terms suggest a development on the part of teachers *is* required, presumably because of the perception that they are not currently in a position to take forward what is being asked of them. This is almost certainly the case in Wales, where the agentic capacity of education professionals to explore new possibilities has been eroded by performative practices embedded over time (Titley et al. 2020). Indeed, Goodson (2007, p. 220) warns that a ‘mixture of profound indifference and active hostility to so many changes’ often negates the personal and professional commitment required of educators to carry out reforms. Given the ‘fatigued’ state in which many Welsh teachers find themselves, this appears a legitimate threat to the successful implementation of *Curriculum for Wales*. Drawing on Freidson’s (2001, p. 127) definition of professional work as that ‘specialized… [and] grounded in a body of theoretically based, discretionary knowledge and skill’, one assumes that new knowledge and skills will have to be acquired by teachers in Wales to effectively discharge their roles in the context of the changing policy landscape. The relationship between this repackaging of the ‘professional sphere’ (Durkheim 1992) within a constantly-evolving reform agenda is a salient one, as it recognises what Goodson describes as the ‘delicate natural ecology’ of teacher professionalism and the ‘sustainable environments’ needed to allow professionalism to flourish (Goodson 2003, p. 74). This habitual inference is further enforced by Wiliam (2016, p. 163), who warns that ‘sustaining changes in what teachers do in their classrooms involves changing highly automated routines’ and that ‘changing practice is essentially a process of habit change’.

Looking ahead, it is important that strategies designed to enable teachers to ‘develop, discuss and practice new knowledge’ in Wales are ‘sustained and intensive rather than brief and sporadic’ (Opfer and Pedder 2011, p. 384). Fullan and Hargreaves (2016, p. 1) take this thinking a step further, arguing that in order to get a sound return on professional learning and development, there must first be an investment. This investment, they submit, begins with attracting teachers with high levels of human capital, whose knowledge, skill and talent is improved and grown gradually through the decisional capital of structured experience and feedback that continuously supports and challenges educators as professionals. They resolve that a culture of *collaborative professionalism* is required, in which teachers ‘develop and grow day by day through feedback and joint work in which student and teacher learning and wellbeing form a mutual feed for the betterment of society’ (ibid, p. 21). An important caveat to Wales’ renewed focus on teacher professionalism is that the concept itself means different things to different people (Fox 1992, p. 2). For example, Ozga’s construction of professionalism as ‘a form of control on the occupation members’ (1995, p.35), that presumably binds its workers to certain rules and regulations, is at odds with the more permissive depiction of teachers as being responsible for forging their *own* career path. Taking the middle ground, Troman (1996, p. 476) resolves that professionalism is not clearly defined and instead ‘a socially constructed, contextually variable and contested concept’ that leaves room for local interpretation. Indeed, in order for teachers in Wales to ‘reprofessionalise’ with a view to designing and developing their own curricula, professional development must be seen as non-threatening and voluntary, as well as demonstrably beneficial. As Ball (2003, p. 218) cautions, ‘the ethics of competition and performance are very different from the older ethics of professional judgment and co-operation’. It is feasible that the cultivation of collaborative professionalism, involving educators from across the country engaging in constructive professional dialogue, could support the development of teachers’ understanding of and confidence in school-level curriculum design and implementation. It must be noted, however, that if we accept professionalism as being socially constructed, with teachers having an integral role in that construction, then so too is it in their gift to accept or resist such undertakings (Helsby 1995, p. 320). That is not to say discourse relating to professionalism is predicated on government’s shaping of it, as has been suggested, and practitioners can instead attempt to ‘mediate demanded or required professionalism with their own agentic modification of it and within differing contexts’ (Evans 2011, p. 862). Indeed, the ‘agentic modification’ of professionalism, however it is manifested, may in fact be necessary if teachers are to adapt successfully to new working arrangements.

Moving forward, it is important that teachers see their own professional learning as being key to the curriculum’s development, and that collaboration with colleagues will support them in this aim. To some, Grundy and Robison's (2004) depiction of professional learning as 'renewal', and that which requires a transformation and change in teachers' knowledge and practice, might be sufficient when articulating the proposed evolution of the education workforce in Wales. But the notion of ‘revitalisation’ is in this context about so much more than knowledge and practice. It is about winning hearts and minds as much, if not more, as it is what teachers know and do – and requires a paradigm shift in the way teachers view and model themselves as naturally curious, energetic and confident drivers of change. That the OECD acknowledged in its progress report on Wales’ reform journey, published in October 2020, that ‘efforts will be needed to help all schools develop the mindset, skills, capabilities and resources to implement the new curriculum’ (OECD 2020, p. 14) serves to reinforce the view that the agentic capacity of teachers is as yet underdeveloped.

## Drawing conclusions

The paper presents a number of key observations based on the last 20 years of education reform in Wales that have major implications for the nation’s education system moving forward. The first relates to the formation of education policy development and its separation into three distinct phases: the initial phase characterised by the freedom to innovate, resistance to high-stakes accountability and giving teachers licence to explore new pedagogical approaches; the second an era dominated by performativity and compliance, prominent in the period immediately following the publication of PISA results in 2010; and more recently, in light of a promised overhaul of Wales’ national curriculum, the beginnings of a more collegiate chapter defined by professionalism and agency. Table 2 provides a brief breakdown of each phase and its associated characteristics for ease of reference.

**Table 2:** The three phases of education policy development in Wales, 1999–2020

Phase	Characteristics
One: Devolution and a licence to innovate (1999–2010)	Comprehensive reform agenda, divergence from England, rowing back from accountability, trust in teachers, innovative policy development
Two: PISA and the age of accountability (2010–2015)	Renewed focus on standards, re-introduction of national testing and school categorisation, stronger emphasis on international comparators, call for data
Three: Curriculum for Wales and a culture of collaboration (2015-)	Whole-system reform, move to self-evaluation, co-construction of policy, collaboration involving range of partners, teacher autonomy

In some respects, the third—and current—phase represents something of a return to the *old* approach to education policy development, in that it resurrects many of the features prominent in Wales during the early 2000s (and prevalent during *Phase One*). Demands on the teaching profession have come full circle, and with greater autonomy comes a newfound sense of optimism that Welsh education is on a promising path to recovery. The education minister’s declaration that made-in-Wales reforms are ‘putting Welsh education on the world map’ (Williams 2018) is testament to a growing confidence, particularly within the political establishment, that the educational course set will pay dividends. The expectation that all teachers in Wales will soon become curriculum designers and, to some extent, creators of their own destiny, adds to the narrative that the nation’s education system is evolving into something markedly different. Indeed, rather than seeking to downplay this transformation, the Welsh Government has actively sought to draw attention to it, as demonstrated by its ‘education is changing’ strapline (see Welsh Government 2020b), though given the almost constant state of flux endured by the country’s educators over the past two decades, ‘education is changing *again*’ might have been a more appropriate slogan. Known challenges with teacher recruitment and retention should be considered in this context.

Further to the changeable educational landscape in Wales, there is another notable observation relating to the evolution of education policy since devolution, namely that associated with the pace at which change has taken place. If the PISA shock of 2010 and publication of *Successful Futures* in 2015 were the stimuli for passage between phases, then there was no discernible ‘policy break’ through which subsequent changes could be properly assimilated. Instead, the transition between Wales’ three phases of reform has been strikingly swift, giving the education system and its central protagonists precious little time to prepare for and adapt to policy revision. This has doubtless taken its toll on a professional workforce known to be suffering the effects of ‘reform fatigue’, although it is ironic that Wales sought to pursue what its own education minister called the ‘biggest set of education reforms anywhere in the UK for over half a century’ (Williams 2019) very shortly after the accusation was first levelled. The challenge of transitioning from a system complicit in the ‘fetishization of data’ (Hardy and Lewis 2017) to one built on professionalism and agency, is compounded by the mistrust in and contempt towards those held responsible by teachers for what they perceive as unwarranted criticism and challenge. Is it widely acknowledged that the performativity culture active in *Phase Two* had a largely demoralising effect on those working on education’s frontlines, with high-stakes external pressures resulting in what Munby and Fullan (2016, p. 3) rightly describe as ‘exhausted, discouraged teachers and leaders, stretched on the rack of contract accountability’. This, in turn, led to a stifling of teacher autonomy and voice (Keddie 2015), and added to the sense of powerlessness emanating from a regime of regulation and prescription. These conditions will have actively eroded the teacher autonomy upon which new reforms are based (Biesta 2004; Priestley 2011; Reeves 2008) and as such, a rebuilding of trust between policymakers and the profession is required.

There is little doubt that the phase sandwiched between those promoting professional autonomy (i.e. *Phase Two*) casts the darkest shadow over Wales’ education system, though the policies themselves were a product of the environment in which they originated. PISA 2009 was a catalyst for change and provided the nation’s newly-appointed minister for education leverage to do as he thought necessary to reverse what was considered at the time to be an alarming trend in performance. Indeed, the performativity discourse prevalent in Wales at the turn of the last decade was arguably not the making of any one politician or event alone, and was instead the result of a longstanding and by no means unwarranted perception that educational standards in Wales were poor relative to others and in need of improvement (Andrews 2014; Evans 2015; Dixon 2016). It is no coincidence therefore that successive Welsh education ministers have resolved that the ‘standards agenda’ remain integral to their wider programme of reform and the need for progress against international benchmarks has been a recurring theme. Despite the clear correlation between standards and the Welsh approach to policy development in the early 2010s, there was likely another motivation for the regressive return to testing and public categorising of schools prevalent during *Phase Two*. Namely, a political incentive on the part of Leighton Andrews, a Welsh Labour minister, to retaliate to the so-called ‘War on Wales’ being waged by the Westminster Government and its Conservative education secretary, Michael Gove (Power 2016). Accusations that the Welsh education system had ‘gone backwards’ under Labour (Gove 2012) were quickly dismissed and the contrasting ideological leanings of both men contributed to a growing hostility between the two nations. Moreover, if we are to accept that ‘fundamentally, policy is about the exercise of political power and the language that is used to legitimate that process’ (Codd 1988, p. 235), then one could argue the case that Andrews’ approach was at least in part borne out of a desire to counter the ‘discourse of derision’ emanating from across the border (Power 2016, p. 11).

## Final thoughts

The evolution of education policy development in Wales since devolution has been far from straightforward and so the separation of the Welsh Government’s approach to education reform into three phases, as outlined in this paper, makes it easier to signpost the key events and policy decisions that have shaped Wales’ education system over time. Moreover, it allows for more considered comparison between approaches to policy development, with a means to better understanding the impact of resulting interventions and their effects on the education workforce.

When taken as a whole, Wales’ education reform story over the past 20 years is one of policy confusion rather than policy coherence, as demonstrated by the contrasting and often contradictory interpretation of accountability as a driver for change. Indeed, this paper supports the view, first presented by the OECD in 2014, that Wales’ education system has been—and remains—susceptible to ‘reform fatigue’ and that the pernicious effects of such policy drift have been felt hardest by teachers. Furthermore, the Welsh experience has demonstrated that political continuity does not necessarily lead to policy congruity, and the preservation of Welsh Labour as the only party to have led in each of the five Senedd Cymru terms has not resulted in the level of stability one might have expected from a dominant administration. There is however some optimism that the crafting of Wales’ new national curriculum, which will not be fully phased in until 2026, embodies a longer-term approach to policy development. The nation’s upturn in the newest iteration of PISA, published in December 2019, offers cause for encouragement as it reduces the likelihood of international rankings being used to justify further radical change. Incremental improvement across all three headline domains was broadly good news for the country’s education system (NFER 2019), which could at last point to PISA progress as evidence of the Welsh reform agenda beginning to take hold. Admittedly, the extent to which advancements in international comparators can be directly attributed to recent policy development is questionable given the known time lag between implementation and outcomes (Schulte 2018), and it is entirely plausible that reforms best associated with policy phases one and two impacted more significantly on Wales’ 2018 PISA scores, than those being currently pursued during *Phase Three*. Moving forward, the forthcoming Senedd Cymru election is perhaps the biggest threat to policy continuity in Wales with commentators predicting ‘unprecedented political turmoil’ involving three main parties jostling for the right to govern (Roderick 2020). Kirsty Williams has already announced her intention to stand down as both minister and member of the Welsh Parliament when votes are cast in May 2021. Assuming that curriculum reform *is* allowed to run its course, benefits will only be realised if it is properly understood and implemented across the country, and teachers are both willing and able to meet its ambitious demands. The transition from what Darling-Hammond (1996, p. 270) coins ‘managing compliance to managing improvement’ is not to be underestimated in this context and, as Wales’ inspectorate has made clear, ‘a stage has now been reached when all schools need to think carefully about what the new curriculum means for them’ (Estyn 2020). Recent changes to school accountability mechanisms will likely help in this regard, in that practitioners will not be wedded to such stringent data collection, albeit a warning from the nation’s chief inspector Meilyr Rowlands about the ‘inconsistency of quality and of approaches to teaching as pupils move through the various phases of education’ (Estyn 2020) serves as a stark reminder of the level of challenge afoot.

Cognisant that ‘teachers are often the last to be heard from on the effects of reform policies and the first to be criticized when reforms fail’ (Elmore and McLaughlin 1988, p. 8), it is imperative that curriculum development is co-owned by policymakers and the professional workforce in Wales, and that trust between both parties is genuine and lasting. Indeed, teachers need to be confident that the reform agenda is a truly shared endeavour and a mutual accountability for curriculum reform is guaranteed. At a school level, there is a wealth of research to suggest that improvement and capacity building are dependent on high levels of relational trust amongst staff (Bryk and Schneider 2002; Earl and Timperley 2008; Kilgore and Reynolds 2011), though it is vital that trust extends beyond the confines of the classroom and permeates the education system more generally.

Together, all of this implies that a fundamental change in approach to education policy reform in Wales is required. More specifically, it is important that policymakers seek to address two things: namely, the instability of policy reform evident since devolution and, simultaneously, the centrality of the teaching profession to both its design and implementation. Indeed, policymakers would do well to give more attention to the impact of policy on the very audience it is almost entirely reliant upon. The paper hereby resolves that successful policy reform in Wales will be dependent on a longer-term vision of what the nation’s education system should look like, as well as the support it provides to its frontline education workforce to deliver on that vision. This chimes with the work of Levin (2012, p. 11), who lists among eight principles of system-wide change ‘a focus on key strategies’, ‘capacity building’ and ‘multi-level engagement’ - principles that are all the more relevant as we respond to and recover from COVID-19.

There is a genuine risk that appreciable deviation from the current reform agenda would turn teacher fatigue into frustration, and a more acute resentment towards policymakers that could jeopardise recent attempts to re-engage the profession in Wales’ *National Mission*. On that basis, the next Welsh Government should consider carefully whether the introduction of a ‘fourth phase’ in policy development is really necessary, bearing in mind that a markedly different approach would have the potential to derail the education system’s pathway to progress yet further. A continuation of the existing change programme would be preferable, given teachers have already invested so heavily in it and the hangover from relentless reform remains a bona fide barrier to new intervention. At the very least, a *back to the future* approach, involving the return to a more neoliberal policy agenda, should be actively avoided if recent momentum is to be maintained.
